# Stedman’s Medical Dictionary

**Published:** 1911-07-15

**Authors:** 


Stedman's Medical Dictionary.—This is a new candidate for the favors of medical and dental practitioners and students. It is edited by Dr. Thomas Lathrop Stedman, A.M., editor of the *Medical Record*, and is published in very good style by Wm. Wood & Co., of New York. Dr. Stedman because of his long editorial career and fine scholarship is not only well qualified, but is in a position to realize the short comings of existing works of this kind, and has undoubtedly made a working book of great value. The new work claims recognition for an unusually complete etymological statement of the derivation of all words as well as their pronounciation. Illustrations are used where helpful in clearing definitions, but not excessively, and the book is not burdened with extensive tables which often render works of this kind unhandy in consulting. The treatment of dental terms is liberal, though not as complete as we could desire. The word prosthodontia is not defined, and prosthesis only as applied to legs and glass eyes. No dental use of obtundent or prophylaxis and many other important words is to be found in the work, showing a lack dental supervision. The dropping of the silent letters of diphthongs has simplified the spelling, but the author hesitates to adopt the idea of dropping the final "e" in spelling chloride, iodine, etc. The book is delightfully printed and bound and comfortable in size, making it a real pleasure to consult.

